# ThyroidNet: A Deep Learning Network for Localization and Classification of Thyroid Nodules

**DOI:** 10.32604/cmes.2023.031229

**Published:** 2023-12-30

**Authors:** Lu Chen, Huaqiang Chen, Zhikai Pan, Sheng Xu, Guangsheng Lai, Shuwen Chen, Shuihua Wang, Xiaodong Gu, Yudong Zhang

**Affiliations:** 1 Ultrasonic Department, Zhongda Hospital Affiliated to Southeast University, Nanjing, 210009, China; 2 School of Physics and Information Engineering, Jiangsu Second Normal University, Nanjing, 211200, China; 3 School of Computing and Mathematical Sciences, University of Leicester, Leicester, LE1 7RH, UK; 4 Department of Information Systems, Faculty of Computing and Information Technology, King Abdulaziz University, Jeddah, 21589, Saudi Arabia; 5 State Key Laboratory of Millimeter Waves, Southeast University, Nanjing, 210096, China; 6 Jiangsu Province Engineering Research Center of Basic Education Big Data Application, Jiangsu Second Normal University, Nanjing, 211200, China; 7 School of Software Engineering, Quanzhou Normal University, Quanzhou, 362000, China; 8 Department of Biological Sciences, Xi’an Jiaotong-Liverpool University, Suzhou, 215123, China

**Keywords:** ThyroidNet, deep learning, TransUnet, multitask learning, medical image analysis

## Abstract

**Aim:**

This study aims to establish an artificial intelligence model, ThyroidNet, to diagnose thyroid nodules using deep learning techniques accurately.

**Methods:**

A novel method, ThyroidNet, is introduced and evaluated based on deep learning for the localization and classification of thyroid nodules. First, we propose the multitask TransUnet, which combines the TransUnet encoder and decoder with multitask learning. Second, we propose the DualLoss function, tailored to the thyroid nodule localization and classification tasks. It balances the learning of the localization and classification tasks to help improve the model’s generalization ability. Third, we introduce strategies for augmenting the data. Finally, we submit a novel deep learning model, ThyroidNet, to accurately detect thyroid nodules.

**Results:**

ThyroidNet was evaluated on private datasets and was comparable to other existing methods, including U-Net and TransUnet. Experimental results show that ThyroidNet outperformed these methods in localizing and classifying thyroid nodules. It achieved improved accuracy of 3.9% and 1.5%, respectively.

**Conclusion:**

ThyroidNet significantly improves the clinical diagnosis of thyroid nodules and supports medical image analysis tasks. Future research directions include optimization of the model structure, expansion of the dataset size, reduction of computational complexity and memory requirements, and exploration of additional applications of ThyroidNet in medical image analysis.

## 1 Introduction

### 1.1 Background and Significance

A thyroid nodule is a localized mass within the thyroid gland that is typically painless and may be benign or malignant. In recent years, the incidence of thyroid nodules has been steadily increasing [1], significantly impacting people’s health and quality of life. Therefore, accurate and timely diagnosis of thyroid nodules and the development of appropriate treatment plans are of paramount importance. Ultrasonography is widely used in clinical practice to detect and evaluate thyroid nodules [2]. However, the localization and classification of nodules still rely on the experience and judgment of medical professionals, leading to potential errors. Therefore, developing highly automated, accurate, and reliable methods for image localization and classification of thyroid nodules is of great clinical value [3].

### 1.2 Clinical Requirements for Thyroid Nodules

Clinical requirements for thyroid nodules [4] can be divided into two main aspects: first, accurate localization of nodules to facilitate measurement of size, shape, and other characteristics to provide evidence for clinical evaluation. Second, accurate classification of nodules, distinguishing between benign and malignant nodules, serves as a reference for devising treatment plans. Traditional image processing methods are challenged by image quality variability and the diversity of nodule shape and density [5]. Therefore, developing novel methods that address the clinical need for thyroid nodule image analysis is essential.

### 1.3 Application of Deep Learning in Medical Image Analysis

Deep learning technology has made significant advances in computer vision, natural language processing [6], and other fields in recent years, particularly in image localization and classification tasks, where it has demonstrated exceptional performance. Deep learning methods overcome the limitations of manually designed features in traditional approaches by automatically learning complex patterns within the data [7]. In medical image analysis, deep learning techniques have achieved remarkable results [8], such as lung nodule detection and skin cancer detection [5]. Applying deep learning to localizing and classifying thyroid nodules in medical image analysis undoubtedly has great potential. Recently, some work has been done on the application of deep learning to thyroid imaging diagnosis by our research group. Our aim is to verify whether our models could automatically locate and classify thyroid nodules, and whether it could achieve the same high level of diagnostic accuracy as that of experienced radiologists.

### 1.4 The Main Contributions of This Paper

The main contributions of this paper are as follows:

(1) In the multitask TransUNet, we propose combining the TransUnet encoder and decoder with multitask learning [9].
(2) We propose DualLoss functions tailored to thyroid nodule localization and classification tasks, balancing the learning of localization and classification tasks to improve the generalization ability of the model [10].
(3) We introduce data augmentation strategies and validate their effectiveness [11].
(4) We submit a novel deep-learning model, **ThyroidNet**, to detect thyroid nodules accurately.
(5) ThyroidNet performs better than other mainstream thyroid nodule localization and classification methods.

In conclusion, we propose a ThyroidNet-based thyroid nodule detection that exploits the advantages of deep learning in medical image analysis and provides robust technical support for clinical diagnosis and treatment.

## 2 Related Works

### 2.1 Localization and Classification of Thyroid Nodule Image

In recent years, significant progress has been achieved in the research of thyroid nodule image localization and classification [12]. Traditional localization and classification methods mainly rely on manually designed features and various pattern recognition techniques, such as template matching [13], clustering algorithms, and machine learning classifiers, such as support vector machines (SVM) and decision trees.

However, these methods face challenges when processing complex thyroid nodule images, such as unstable image quality and diversity in nodule shape and density [14]. To address these issues, researchers have begun exploring deep-learning methods to facilitate thyroid nodule image localization and classification performance. Some research groups also made progress on the application of deep learning algorithms to thyroid ultrasound cancer diagnoses [15], and their experimental results achieved a high level of accuracy compared with radiologist’s manual identification [16,17].

### 2.2 Deep Learning Models in Localization and Classification

Deep learning models, particularly convolutional neural networks (CNNs), have shown excellent performance in image localization and classification tasks [18]. Among them, deep networks such as VGG and ResNet have achieved remarkable results in classification tasks. In contrast, models such as YOLO (You Only Look Once) and SSD (Single Shot MultiBox Detector) have shown their effectiveness in object localization [19].

In medical image analysis, these deep learning models have been extensively applied to tasks such as lung nodule localization and classification and other medical image analysis applications [20]. The paper [15] proposed a kind of ensemble deep learning-based classification model (EDLC-TN) for precise thyroid nodules localization. The work [16] developed and trained a deep CNN model called the Brief Efficient Thyroid Network (BETNET) using 16,401 ultrasound images and demonstrates the general applicability. Then a multiscale detection network for classifying thyroid nodules was proposed with an attention-based method [17]. By exploiting the strength of these models, researchers aim to develop more accurate and efficient methods for localization and classification tasks in medical image analysis.

### 2.3 TransUnet

TransUnet is an innovative deep-learning architecture that combines the advantages of Transformer and U-Net models, specifically designed for thyroid nodule localization and classification in medical image analysis [21]. This architecture consists of encoder and decoder modules, where the encoder extracts high-level features from the input image combined with convolutional and transformer layers.

The convolutional layers in TransUnet focus on capturing local information using local receptive fields, allowing the model to learn fine-grained details of the thyroid nodules. This allows TransUnet to capture medical images’ intricate features and subtle patterns effectively [22].

What sets TransUnet apart is the integration of Transformer layers, which excel at capturing long-range dependencies and global contextual information through their self-attention mechanism [23]. This mechanism allows the model to consider relationships between spatially distant regions of the input image, resulting in a more comprehensive understanding of the overall structure and context of the image [24].

By combining local and global feature extraction, TransUnet enhances its ability to localize and classify thyroid nodules accurately, overcoming the limitations of traditional methods that rely solely on local features. This capability is significant in medical image analysis, where thyroid nodules exhibit shape, density, and image quality variations.

Integrating Transformer and U-Net into TransUnet provides a powerful framework for accurately analyzing thyroid nodules. The U-Net-like decoder maps the high-level features back into the original image space, enabling pixel-level segmentation and precise localization of thyroid nodules [25]. Combining convolutional and transformer layers allows TransUnet to capture local detail and global context, improving segmentation and localization performance.

### 2.4 Multitask Learning in Medical Image Analysis

Multitask learning has emerged as a promising approach in the field of medical image analysis, aiming to improve the performance and generalization of deep learning models by jointly optimizing multiple related tasks [26]. By exploiting the inherent relationships between tasks, multitask learning can effectively address the challenges posed by limited labeled data and complex dependencies between medical image analysis tasks [27].

There are often multiple tasks of interest in medical image analysis, such as segmentation, classification, and localization [28]. Traditionally, these tasks have been treated as separate and independent problems, leading to suboptimal performance and limited knowledge transfer between tasks. On the other hand, multitask learning provides a solution by enabling the model to learn shared representations that capture task-specific and shared information [29].

By learning multiple tasks together, the model can benefit from the complementary information in the data, leading to improved performance on each task. For example, in thyroid nodule analysis, the nodule localization and classification tasks are closely related. Accurate localization is critical for accurate classification and vice versa. Multitask learning allows the model to exploit the interdependencies between these tasks, leading to improved performance in both localization and classification [30].

In addition, multitask learning offers the advantage of improved generalization. By learning from multiple related tasks simultaneously, the model can better capture the underlying patterns and structures in the data, resulting in improved performance on unseen samples [31]. This is particularly valuable in medical image analysis, where labeled data is often limited, and acquiring new labeled samples can be challenging.

Several strategies can be used to implement multitask learning effectively. One common approach is to share the initial layers of the network across tasks, allowing the model to learn common representations [29]. This facilitates knowledge transfer between tasks and promotes the discovery of task-specific features in subsequent layers.

Appropriate loss functions and regularization techniques can be used to balance the learning process across tasks. This ensures that the model does not favor one task over the other and achieves a good trade-off between task-specific and shared representations.

Overall, multitask learning holds great promise for medical image analysis tasks. Jointly optimizing multiple tasks allows the model to exploit the relationships and dependencies between them, improving performance and generalization. In the context of thyroid nodule analysis, multitask learning can improve localization, classification, and other related tasks, ultimately providing more accurate and reliable diagnostic capabilities for medical professionals [32].

## 3 Methodology of Proposed ThyroidNet

### 3.1 Datasets and Preprocessing

#### 3.1.1 Source and Composition of the Dataset

**Figure 1:**
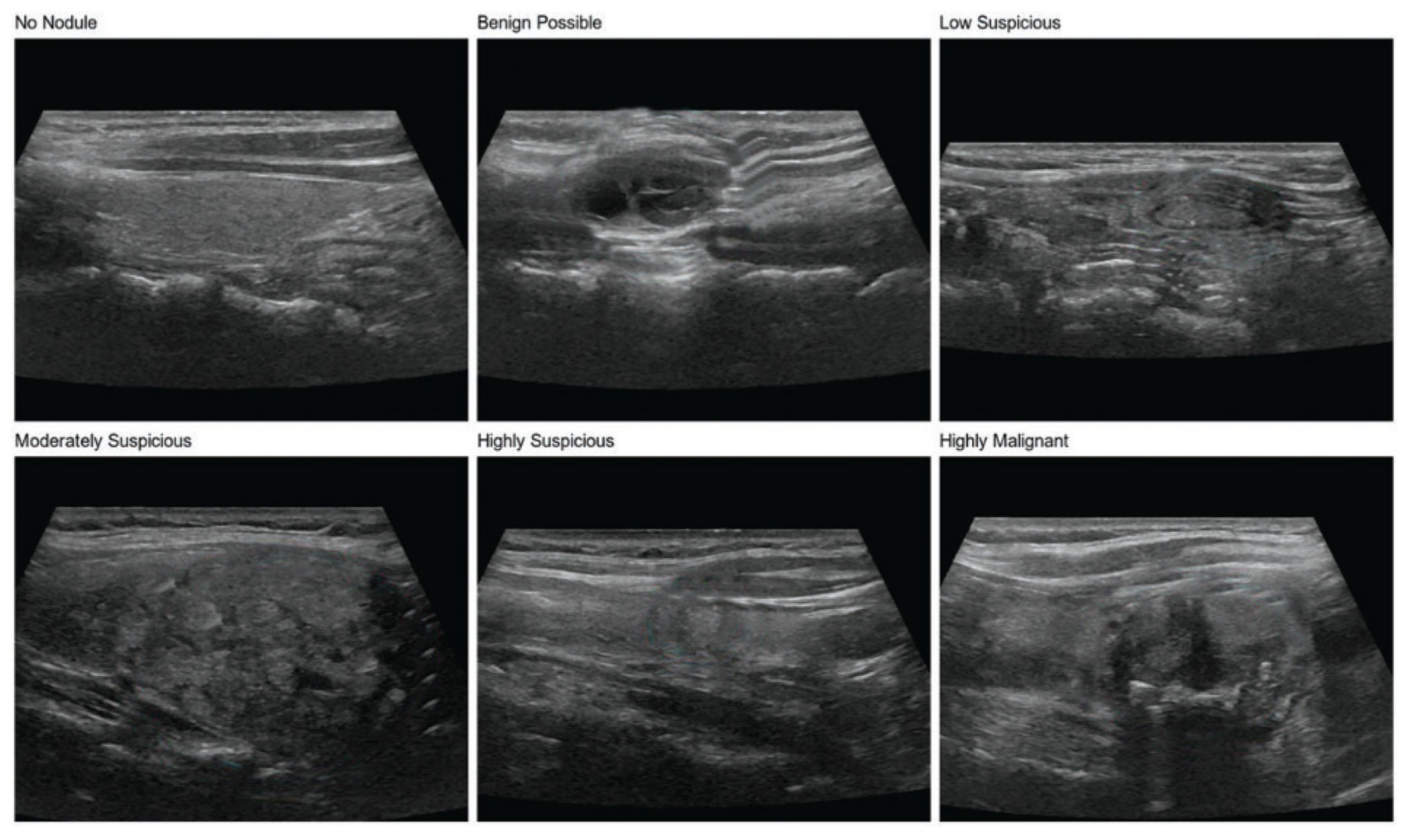
Six types of thyroid nodules in the dataset

The thyroid nodule dataset used in this study is a private dataset derived from ultrasound images provided by the ultrasound department of Zhongda Hospital, affiliated with Southeast University, Nanjing, Jiangsu, China. The judgment of patient images is done by professional radiologists. The dataset contains 600 thyroid nodule images, covering six categories of a thyroid nodule, including no nodule, benign possible, low suspicious, moderately suspicious, highly suspicious, and highly malignant, as shown in Fig. 1, with 100 images for each type of nodule. In addition, the dataset includes nodules of different shapes, sizes, and densities, which is conducive to training models with a strong generalization ability to meet the needs of practical clinical applications in the hospital.

#### 3.1.2 Data Preprocessing

Data preprocessing is essential in training ThyroidNet to improve the model’s effectiveness. In this study, several preprocessing methods were employed to ensure the quality and suitability of the dataset for training the deep learning model.

**Normalization:** Normalization is used to standardize pixel values to account for variations in brightness and contrast between images. By dividing each pixel value by 255, the pixel values are rescaled to a range between 0 and 1. This normalization process eliminates differences in image intensities, allowing the model to concentrate on the relevant features of the thyroid nodules rather than being influenced by intensity variations.

**Cropping:** Cropping is performed on the original images to reduce computational complexity and memory requirements without compromising the integrity of the nodules [33]. A fixed size of 256 × 256 pixels is chosen for the cropped images in this study. The cropping area is determined based on the nodule boundary boxes, ensuring the nodules are centered within the cropped images. This cropping strategy helps to standardize the input size and allows the model to focus on the region of interest, facilitating accurate localization and classification of the nodules.

Data pre-processing plays a crucial role in preparing the dataset for training ThyroidNet. The normalization step reduces the impact of intensity variations, allowing the model to learn meaningful patterns and features. Cropping images to a fixed size reduces computational complexity and ensures that nodules are prominently represented in the input data. These pre-processing techniques contribute to the overall performance of ThyroidNet by improving its ability to diagnose thyroid nodules accurately.

### 3.2 Multitask TransUNet

To address thyroid nodules’ localization and classification tasks, we propose a multitask learning approach using a modified version of the TransUNet architecture. Our model, named ThyroidNet, combines the strengths of TransUNet for localization with an additional classification branch, allowing simultaneous learning of features relevant to both tasks and improving overall performance.

In the proposed multitask TransUNet, we exploit the encoder-decoder architecture of TransUnet. The encoder module extracts high-level features from the input image using a combination of convolutional and transform layers. These layers allow ThyroidNet to extract local and global information for accurately localizing and classifying thyroid nodules. The encoder module is formulated as follows (1):

(1) $$\{\begin{array}{l} h_{i}^{l}=ConvLayer\left(h_{i}^{l-1}\right)\\ z_{i}^{l}=MHSA\left(h_{i}^{l}\right)\\ h_{i}^{l}=LayerNorm\left(z_{i}^{l}+h_{i}^{l-1}\right) \end{array}$$

Where $h_{i}^{l}$ the *i*-th encoder layer output at level l, ConvLayer denotes the convolutional layer, MHSA refers to the Multi-Head Self-Attention mechanism, and LayerNorm represents the layer normalization operation.

In the decoder module, we introduce two separate branches: one for nodule localization and the other for nodule classification. The localization branch aims to generate precise spatial maps highlighting the exact locations of thyroid nodules within the input image. The classification branch, on the other hand, focuses on assigning labels to the detected nodules.

The localization branch is formulated as follows (2):

(2) $$\{\begin{array}{l} d_{i}^{l}=UpConv\left(d_{i+1}^{l+1}\right)=LayerNorm\left(z_{i}^{l}+d_{i+1}^{l+1}\right)\\ a_{i}^{l}=Concat\left(h_{i}^{l},d_{i}^{l}\right)\\ z_{i}^{l}=MHSA\left(a_{i}^{l}\right) \end{array}$$

where $d_{i}^{l}$ represents the output of the *i*-th decoder layer at level *l*, UpConv denotes the up-sampling convolutional layer, and Concat represents the concatenation operation.

The classification branch is formulated as follows (3):

(3) $$\{\begin{array}{l} c_{i}^{l}=UpConv(c_{i+1}^{l+1})=LayerNorm(y_{i}^{l}+c_{i+1}^{l+1})\\ b_{i}^{l}=Concat(h_{i}^{l},c_{i}^{l})\\ y_{i}^{l}=ConvLayer(b_{i}^{l}) \end{array}$$

Where $c_{i}^{l}$ represents the output of the *i*-th classification layer at level *l*.

The shared encoder parameters facilitate knowledge transfer between the localization and classification tasks, allowing the model to learn robust representations that capture task-specific and shared information. This sharing of encoder parameters increases learning efficiency and enables the model to exploit the complementary information present in the data [34].

By jointly optimizing both tasks within a single model, ThyroidNet can exploit the interdependencies between localization and classification, improving performance in both tasks. The shared encoder architecture enables the model to learn rich, discriminative features that benefit localization and classification.

Furthermore, the decoder module introduces a novel attention mechanism called Multi-Head Self-Attention (MHSA) [35] within the decoder module. MHSA allows ThyroidNet to pay attention to different regions of the input image simultaneously, thereby improving localization and capturing fine-grained details of thyroid nodules. This attention mechanism enhances the model’s ability to focus on informative regions while suppressing irrelevant background regions, thereby improving overall performance.

### 3.3 Proposed DualLoss Function

The DualLoss function is a crucial component in ThyroidNet, designed to strike a balance between the learning of localization and classification tasks, thereby enhancing the model’s generalization ability. It is achieved by combining two separate loss functions, one for each task: the localization loss (L_loc) and the classification loss (L_class) [36].

**Localization Loss (L_loc):** The localization loss is a critical metric for evaluating the model’s performance in accurately identifying the spatial position of thyroid nodules within medical images. It quantifies the discrepancy between the ground truth localization map, which represents the true locations of thyroid nodules, and the predicted localization map, which indicates the model’s estimation of nodule locations. The Dice loss function is a widely-used pixel-wise loss metric for this purpose (4).

(4) $$\{\begin{array}{l} L\text{\_}loc=1-DSC(A,B)\\ DSC(A,B)=\frac{2*|A\cap B|}{\left|A|+|B\right|} \end{array}$$

This loss function effectively captures the model’s performance in localizing thyroid nodules within medical images by measuring the agreement between the predicted and ground truth localization maps [37]. ThyroidNet aims to improve its ability to accurately localize thyroid nodules in medical images by optimizing this loss during training.

**Classification Loss (L_class):** The classification loss evaluates the model’s performance in correctly identifying the category of thyroid nodules in the images. It measures the dissimilarity between the ground truth labels and the predicted class probabilities. The multi-class cross-entropy loss is a widely used loss metric for multi-class classification problems. In this case, the classification loss is combined with the Dice loss function to form a modified loss function (5).

(5) $$L\text{\_}class=\frac{1}{N}*\sum_{i=1}^{N}\left(\sum_{j=1}^{C}\left(y_{ij}*log\left(p_{ij}\right)*l\text{\_}loc+\left(1-y_{ij}\right)*log\left(1-p_{ij}\right)*\left(1-L\text{\_}loc\right)\right)\right)$$

Here, L_class denotes the modified classification loss, L_loc represents the Dice loss function for localization, and N is the total number of samples. The variables *y*_*ij*_ and *p*_*ij*_ stand for the true label for the *j*-th class of the *i*-th sample (1 if the true class, 0 otherwise) and the predicted probability for the *j*-th class of the *i*-th sample, respectively. The outer summation ∑ iterates over all samples in the dataset (from i = 1 to N), while the inner summation ∑ iterates over all the classes in the problem (from j = 1 to C).

Incorporating the Dice loss function, the modified multi-class cross-entropy loss considers the model’s classification and localization performance [38]. This joint loss function evaluates the model’s ability to classify thyroid nodules accurately while considering the localization information. ThyroidNet aims to improve its performance in classifying and localizing thyroid nodules within medical images by minimizing this combined loss during training.

**DualLoss:** The DualLoss Function combines the Localization Loss (L_loc) and the modified Classification Loss (L_class). It is designed to optimize both localization and classification tasks simultaneously during the training process of ThyroidNet. The DualLoss Function can be defined as follows (6).

(6) DualLoss=α∗L_loc+β∗L_class

In this equation, *α* and *β* are weight parameters that balance the contributions of the localization loss (L_loc) and the modified classification loss (L_class) to the overall DualLoss Function. By minimizing the DualLoss Function, ThyroidNet aims to improve its performance in accurately localizing and classifying thyroid nodules in medical images. The model adjusts the weight parameters *α* and *β* according to the application’s requirements, ensuring an optimal balance between localization and classification performance [39].

### 3.4 Data Augmentation Strategy

In order to further enhance the model’s generalization ability, we adopt a data augmentation strategy [11]. Data augmentation generates new training samples through various image transformations, thus enlarging the scale of the training set. In this study, we used the following data augmentation methods:

**Rotation:** The image is rotated at random angles, ranging from −15 to 15 degrees. This helps the model adapt to nodular images from different angles.

**Translation:** The image is translated horizontally and vertically at random with a range of 5% of the image width and height. This helps the model adapt to different positions of the nodules in the image.

**Zoom:** The image is randomly scaled with a range of 0.9 to 1.1 times. This helps the model adapt to nodules of different sizes.

**Flip:** The image is flipped horizontally. This helps the model adapt to nodules in diverse directions [40].

**Contrast and brightness adjustment:** The image is randomly adjusted for contrast and brightness, varying from 0.8 to 1.2 times. This helps the model adapt to images with different contrast and brightness [41].

**Figure 2:**
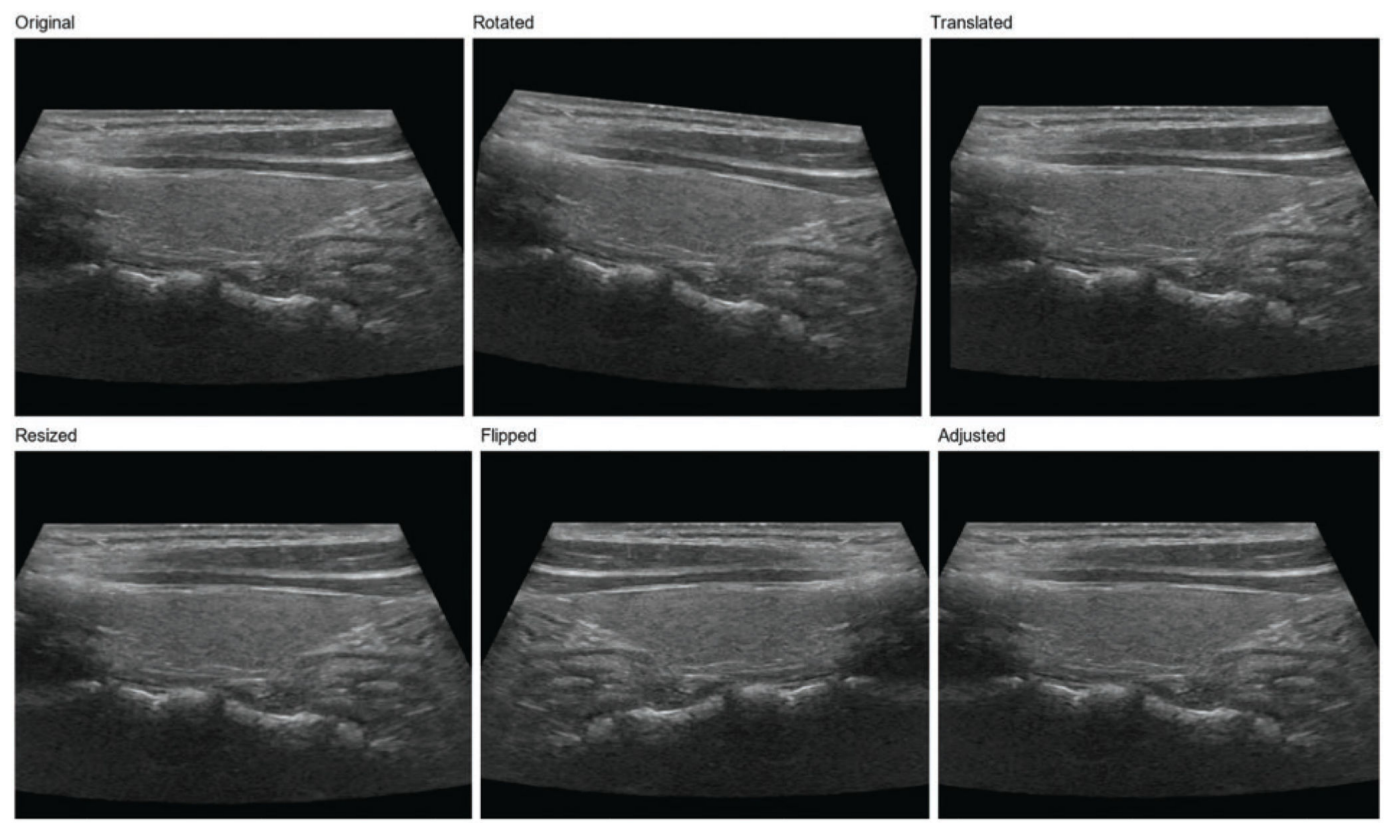
Thyroid nodule image enhancement

Using these data enhancement methods, as shown in Fig. 2, we can significantly improve the model’s generalization ability and enhance its performance when handling actual clinical data. Simultaneously, data augmentation also is used to reduce overfitting and improve model performance on validation and test sets [42]. In the subsequent experiments, we will evaluate the effects of numerous data augmentation methods on ThyroidNet’s performance to select the best augmentation strategy.

### 3.5 Architecture of ThyroidNet

**Figure 3:**
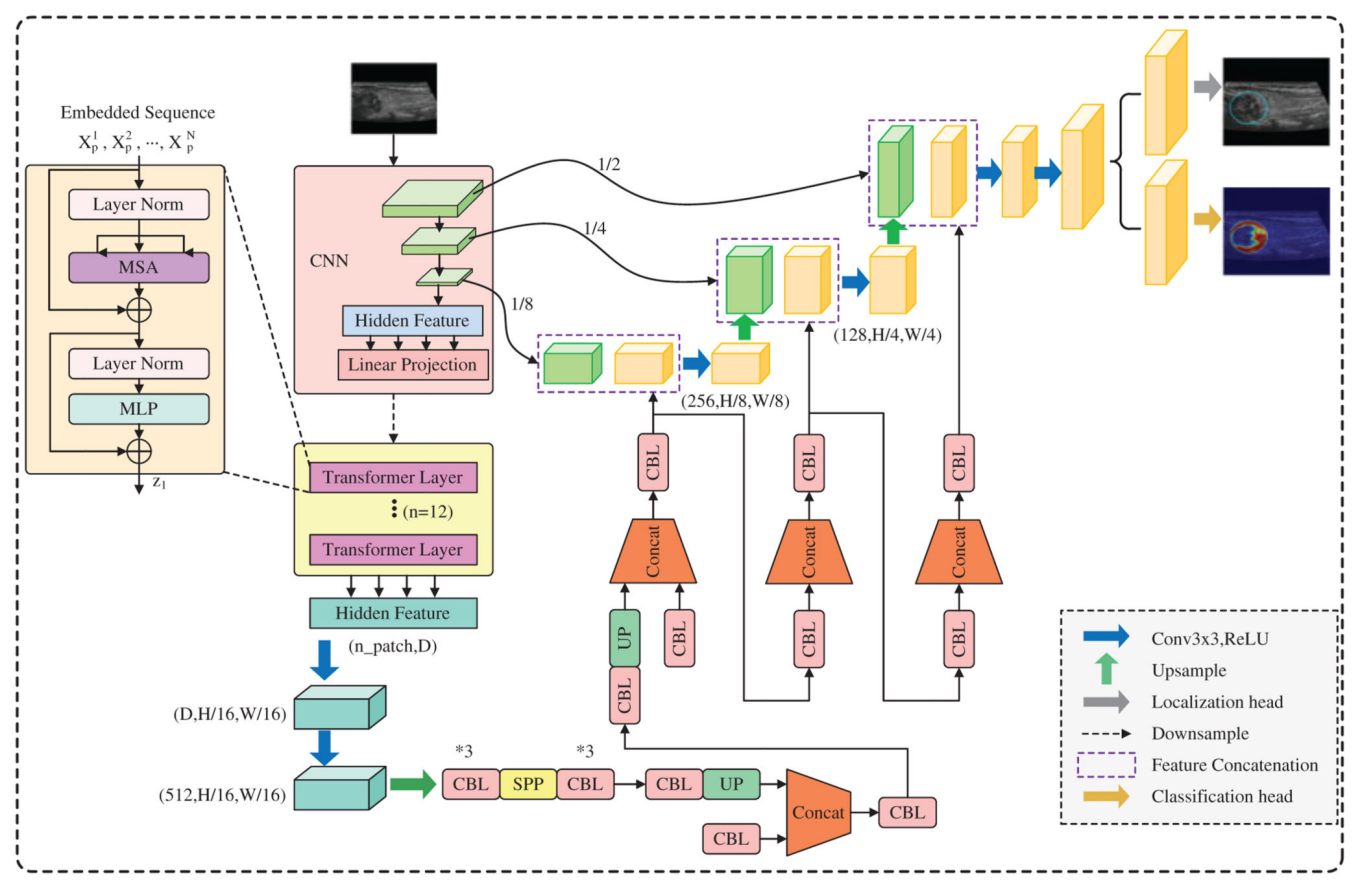
ThroidNet neural network architecture diagram

ThyroidNet’s specific structure is shown in Fig. 3. ThyroidNet is an advanced deep learning architecture designed to localize and accurately classify thyroid nodules in medical images. By leveraging the strengths of TransUnet, the model effectively captures local and global features to enhance its performance [43]. Multitask Learning is employed to optimize localization and classification tasks simultaneously, allowing the model to learn features relevant to both tasks concurrently [44].

The DualLoss function, combining Localization Loss (L_loc) and the modified Classification Loss (L_class), plays a crucial role in balancing learning localization and classification tasks. This approach ensures the optimization of both tasks, leading to improved performance in accurately localizing and classifying thyroid nodules in medical images.

By refining its performance in localizing and classifying thyroid nodules, ThyroidNet has the potential to significantly contribute to advancing medical imaging and diagnosis in the field of thyroid disease.

The ThyroidNet is an innovative deep learning-based method developed for thyroid nodule localization and classification tasks. Its design is motivated by the need to address the challenges associated with the efficient and accurate detection of thyroid nodules in medical images. The key components of ThyroidNet’s neural network structure are as follows:

Integration of TransUnet: ThyroidNet leverages the TransUnet architecture, which combines the Transformer and U-Net models. The Transformer architecture, originally introduced for natural language processing tasks, has shown remarkable capabilities in capturing long-range dependencies and learning contextual information. By incorporating Transformer blocks into the U-Net, ThyroidNet can effectively model global context while retaining the excellent feature extraction capabilities of U-Net for image segmentation.

Multitask Learning: To unify the localization and classification tasks and improve the overall performance of ThyroidNet, multitask learning is employed. The network is trained jointly to simultaneously perform both tasks, with shared feature representations. This enables the model to leverage the complementary information from localization and classification tasks, leading to enhanced detection and better classification of thyroid nodules.

Data Augmentation Strategies: ThyroidNet utilizes various data augmentation techniques to augment the training dataset. Augmentation includes image rotations, flips, zooming, and other transformations, which help improve the model’s ability to generalize to diverse real-world clinical data. This ensures that ThyroidNet remains robust and effective even when dealing with variations in image appearances and nodule characteristics.

DualLoss Function: The loss function used in ThyroidNet, referred to as DualLoss, is designed to handle both localization and classification tasks jointly. The DualLoss combines the Dice loss, which is well-suited for segmentation tasks, and the cross-entropy loss, commonly used in classification tasks. This combination enables ThyroidNet to effectively optimize the model for both tasks simultaneously, striking a balance between accurate localization and precise classification.

### 3.6 Experimental Design

#### 3.6.1 Experimental Settings

**Hardware environment:** We experimented on a computer equipped with an NVIDIA GeForce RTX 3090 graphics card, 64 GB of RAM, and an Intel Core i9-10900K processor.

**Software environment:** We used the Python programming language for model implementation, using the PyTorch deep learning framework. We also used the OpenCV library for image processing and the Scikit-learn library for evaluation metrics.

**Training parameters:** When training ThyroidNet, we adopted the following parameter settings: a learning rate of 10^−4^, a batch size of 64, and 500 training epochs.

#### 3.6.2 Selection of Comparative Methods

To evaluate the performance of ThyroidNet, which integrates TransUnet for localization and multitask learning for the classification of six categories of thyroid nodules (including five positive categories), we selected the following comparison methods:

**U-net-based method:** Comparing ThyroidNet with the classic U-net allowed us to evaluate the impact of incorporating TransUnet and multitask learning [17].

**TransUnet-based method:** We compared ThyroidNet with the original TransUnet to evaluate the effect of incorporating multitask learning for classification tasks [19].

**Traditional image segmentation methods:** ThyroidNet was also compared with traditional methods based on threshold segmentation to illustrate the superiority of deep learning methods in thyroid nodule segmentation and classification tasks.

In addition, we compared the performance of ThyroidNet and the following models on the datasets in this experiment:

**FCN:** ThyroidNet is compared with FCN as a benchmark model to assess the improvements achieved by incorporating TransUnet and multitask learning for thyroid nodule localization and classification tasks [45].

**Mask R-CNN:** Mask R-CNN is compared with ThyroidNet to explore the advantages and disadvantages of instance-level segmentation methods for thyroid nodule localization and classification [46].

**SegNet:** SegNet and ThyroidNet are compared to investigate the impact of different model architectures on thyroid nodule localization and classification tasks [47].

**DeepLab:** DeepLab is another model to compare with ThyroidNet to investigate the benefits of multiscale feature extraction for thyroid nodule localization and classification [48].

Finally, we tested the performance of ThyroidNet on different datasets to thoroughly evaluate its applicability and effectiveness in different scenarios.

#### 3.6.3 Evaluation Metrics

We used the following metrics to evaluate the performance of the model in the thyroid nodule localization and classification tasks [49]:

**Dice:** The Dice coefficient measures segmentation quality ranging from 0 to 1. A higher Dice coefficient indicates more agreement between segmentation results and ground-truth annotations.

**Accuracy:** Accuracy is an evaluation metric for classification tasks, representing the proportion of samples correctly classified by the model out of the total number of samples.

**Precision:** Precision is an evaluation metric for classification tasks and represents the proportion of true positive cases correctly identified by the model out of all samples identified as positive cases by the model.

**Recall:** Recall is an evaluation metric for classification tasks and represents the proportion of true positive cases correctly identified by the model out of all confirmed positive cases (covering five categories of thyroid nodules).

**F1:** The F1 score is the harmonic mean of precision and recall and is used to evaluate the classification model’s performance comprehensively. The F1 score ranges from 0 to 1, with values closer to 1 indicating better model performance.

In this study, we will separately calculate the performance metrics of ThyroidNet for different tasks, including the Dice coefficient, accuracy, precision, recall, and F1 score. At the same time, we will compare ThyroidNet with various control methods to comprehensively evaluate the performance of ThyroidNet. Through the above experimental design and evaluation metrics, we aim to explore in depth the performance of ThyroidNet in the localization and classification of thyroid nodules and provide further validation of its potential value in clinical applications. In addition, this study will provide valuable insights and guidance for future research in related areas.

By incorporating TransUnet for localization and multitask learning for classification, we aim to provide an efficient and accurate thyroid nodule localization and classification method. The experimental design and evaluation metrics used in this study will help validate the performance and potential value of ThyroidNet in clinical applications, providing valuable insights and guidance for researchers and medical professionals working in thyroid nodule detection and related areas. By comparing ThyroidNet with various control methods and models, we will highlight the benefits of integrating TransUnet and multitask learning in thyroid nodule localization and classification tasks.

## 4 Experiments

### 4.1 Comparison of Localization and Classification Performance of ThyroidNet

Table 1 compares different models’ localization and classification performance, including ThyroidNet, U-Net, TransUnet, traditional image segmentation methods, FCN, Mask R-CNN, SegNet, and DeepLab. The evaluation metrics used for comparison include the Dice coefficient, accuracy, precision, recall rate, and F1 score [19,50,51].

It is important to note that the reported performance of each model in Table 1 is contingent upon their specific training methodologies, with variations in the utilization of augmented data during training. However, ThyroidNet, in particular, achieved remarkable results by incorporating TransUnet and multitask learning. This led to significant improvements in the Dice coefficient, accuracy, precision, recall rate, and F1 score compared to U-Net, TransUnet-based, and traditional image segmentation methods.

ThyroidNet demonstrated a Dice coefficient of 0.957, accuracy of 0.950, precision of 0.984, recall rate of 0.971, and an F1 score of 0.976. In contrast, U-Net, TransUnet, traditional image segmentation methods, FCN, Mask R-CNN, SegNet, and DeepLab achieved lower scores across these metrics. These results indicate the superior performance of ThyroidNet in localizing and classifying thyroid nodules.

Moving on to Table 2, it compares the localization and classification performance on different datasets. ThyroidNet consistently exhibited excellent performance across various datasets. Moreover, ThyroidNet achieved a comparable or even better performance than other models on each dataset, as reflected by the Dice coefficient, accuracy, precision, recall rate, and F1 score.

The results presented in Tables 1 and 2 demonstrate the outstanding performance of ThyroidNet. By incorporating TransUnet and multitask learning, ThyroidNet outperformed other models regarding the localization and classification of thyroid nodules. These findings emphasize the effectiveness of the proposed methodology and highlight the suitability of ThyroidNet for tasks involving thyroid nodule localization and classification.

### 4.2 Performance of the Model under Different Data Augmentation Strategies, Network Structure Adjustments, and Loss Function Design

We further investigated the performance of ThyroidNet, now based on TransUnet and multitask learning for thyroid nodule localization and classification, under different data augmentation strategies, network structure adjustments, and loss function designs. Table 3 shows the performance comparison for these scenarios:

The generalization ability of ThyroidNet is significantly improved when the data augmentation strategy is applied, as evidenced by the increase in the Dice coefficient, accuracy, precision, recall, and F1 score compared to the model without data augmentation.

In addition, when trying different network structure adjustments, we found that combining TransUnet with multitask learning (MT) further optimized model performance, leading to better evaluation metrics.

Finally, we investigated the effects of discrete loss function designs on model performance. We found that an appropriate loss function design (DualLoss: (7)) can help the model better balance localization and classification tasks, resulting in higher evaluation metrics than the alternative loss function (OriginalLoss: (7)).

(7) $$\{\begin{array}{l} L_{-}loc=1-\frac{2*|A\cap B|}{\left|A|+|B\right|}\\ OriginalLoss=\alpha *L\text{\_}loc+\beta *\left(-\frac{1}{N}*\sum_{i=1}^{N}\left(\sum_{j=1}^{C}\left(y_{ij}*log\left(p_{ij}\right)+\left(1-y_{ij}\right)*log\left(1-p_{ij}\right)\right)\right)\right)\\ DualLoss=\alpha *L\text{\_}loc+\beta *\left(-\frac{1}{N}*\sum_{i=1}^{N}\left(\sum_{j=1}^{C}\left(y_{ij}*log\left(p_{ij}\right)*L_{-}loc+\left(1-y_{ij}\right)*log\left(1-p_{ij}\right)*\left(1-L_{-}loc\right)\right)\right)\right) \end{array}$$

These findings support the importance of selecting appropriate data enrichment strategies, network structure adjustments, and loss function designs to optimize the performance of ThyroidNet in thyroid nodule localization and classification.

### 4.3 Performance Differences of the Models in Different Categories of Thyroid Nodules

We also assessed the performance differences of the models for different categories of thyroid nodules. Table 4 shows that ThyroidNet has a high recognition ability across distinct categories of thyroid nodules. However, the model’s performance slightly decreased in some categories, such as nodules with lower contrast or smaller size. This suggests that ThyroidNet still has room for improvement when dealing with these challenging nodules.

### 4.4 Visual Presentation of Model Performance

**Figure 4:**
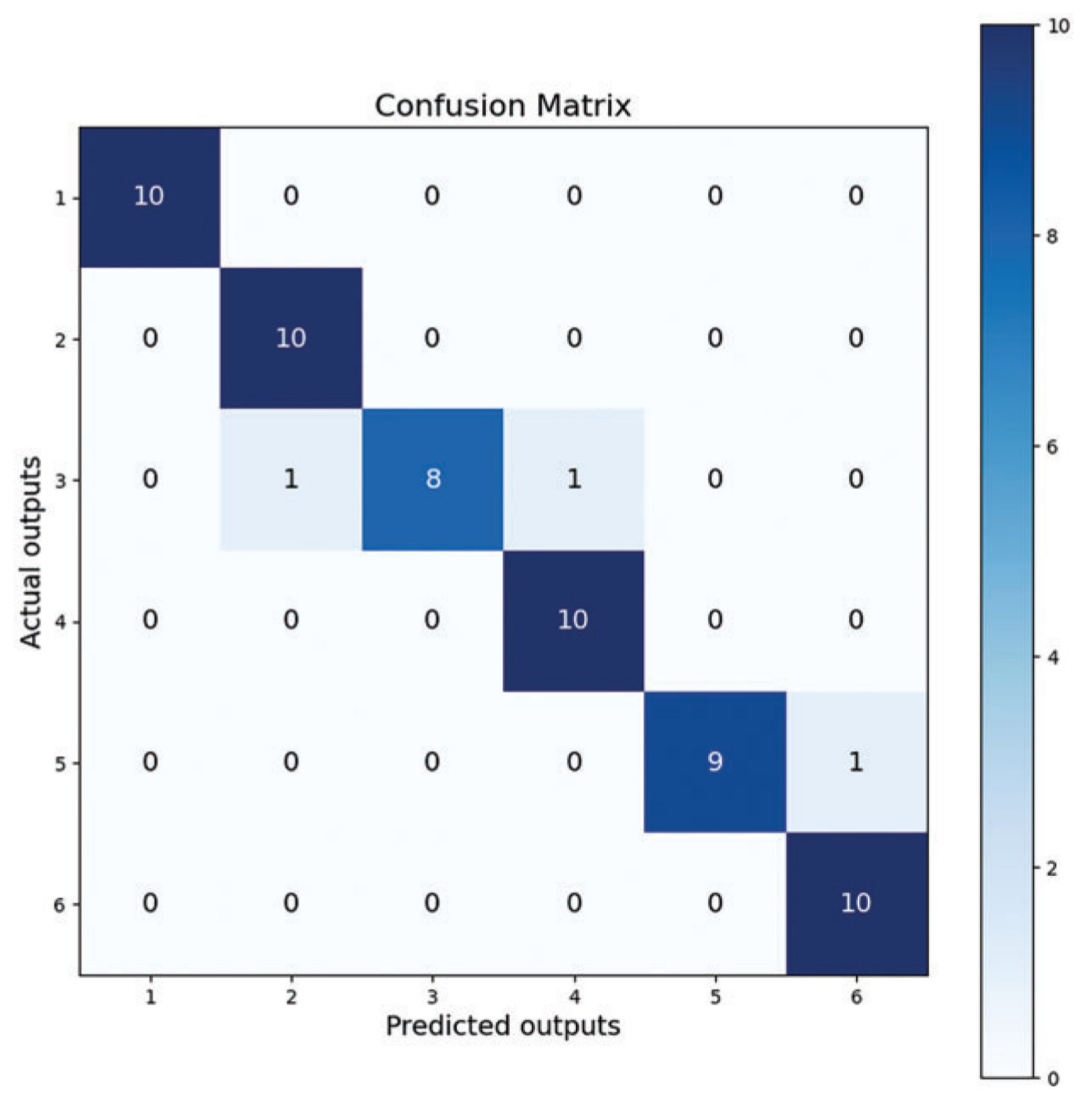
Confusion matrix

We used the confusion matrix visualization method to demonstrate the model’s performance [52]. The performance of ThyroidNet on the classification tasks of different thyroid nodules can be seen from the confusion matrix in Fig. 4. The confusion matrix shows that most nodules were correctly classified, and only a few were misclassified. This proves that the model has a high classification accuracy.

In conclusion, ThyroidNet performs well in the localization and classification of thyroid nodules. ThyroidNet significantly improves the evaluation metrics compared to other methods. Experiments with different data augmentation strategies, network structure adjustments, and loss function designs show that the model has a better generalization ability and performance optimization potential [53]. Although the model’s performance decreased slightly on certain difficult nodes, the overall performance was still very satisfactory. The visualization of the confusion matrix further confirmed the superior performance of ThyroidNet in the segmentation and classification of thyroid nodules.

Regarding the numbers in Fig. 4, it is crucial to explain the sum of values in each row being 10, as it relates to the composition of our dataset. Our dataset consists of six distinct types of thyroid nodules, with 100 images per type, and the test set constitutes ten percent of the entire dataset. Therefore, the confusion matrix reflects these proportions accurately.

**Figure 5:**
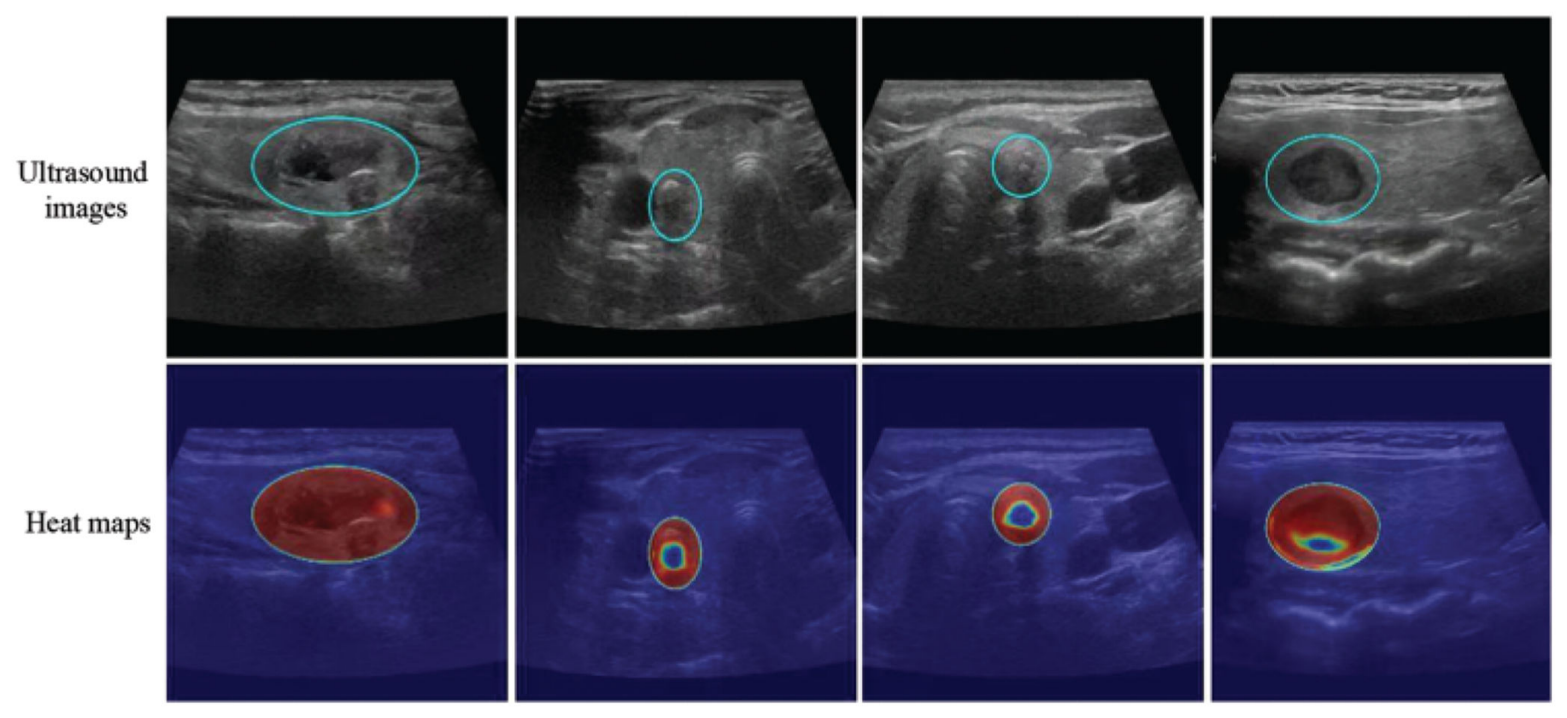
Localization of thyroid nodules. The blue circle is the location of the thyroid nodules, and the heat map represents the visualization results. Each column shows the same ultrasound image. The cold tone region of the visualization image is the most important part that our model could recognize

The model successfully focused on the thyroid nodule areas in the ultrasound images. As seen in Figs. 4 and 5, the blue box area denotes the thyroid nodule location, and the heat map represents the visual location result.

## 5 Discuss and Future Work

### 5.1 The Potential Value of ThyroidNet in Clinical Application

ThyroidNet demonstrates strong performance in the localization and classification of thyroid nodules with high accuracy. This gives ThyroidNet significant potential value in clinical applications. By automatically and accurately identifying and classifying thyroid nodules, ThyroidNet helps reduce the risk of misdiagnosis and missed diagnoses and improves the effectiveness of clinical diagnosis [54]. In addition, ThyroidNet can help clinicians develop more appropriate treatment plans, thereby improving patient outcomes and quality of life.

### 5.2 Compare the Pros and Cons of Other Methods

ThyroidNet has several advantages over U-Net, TransUnet-based methods, and traditional image segmentation methods:

Applying TransUnet and multitask learning improves the model’s handling of thyroid nodule localization and classification tasks.

Using data enhancement strategies improves the model’s generalization ability, making it more suitable for actual clinical data processing.

Proper loss function design allows the model to balance localization and classification tasks, improving overall performance [55].

However, other methods have advantages in certain aspects. For example, traditional image segmentation methods are more suitable for resource-constrained scenarios due to their low computational complexity and memory requirements [56].

### 5.3 Limitations and Challenges of the Method

Despite the remarkable performance of ThyroidNet in the tasks of localization and classification of thyroid nodules using TransUnet and multitask learning, some limitations and challenges remain:

The model’s performance slightly decreases when dealing with low-contrast or small nodules, suggesting that ThyroidNet has room for improvement when dealing with these challenging nodules.

Limited by the training dataset size, the model may need to be able to generalize sufficiently when confronted with more complex and diverse clinical data [57].

ThyroidNet has relatively high computational complexity and memory requirements, which may make it unsuitable for certain resource-constrained scenarios [58].

### 5.4 Future Research Directions and Applications

Considering the above limitations and challenges, future research directions and applications can be explored from the following perspectives:

**Optimization of the model structure:** The model structure can be improved to improve the performance on low contrast or small-size nodules. For example, introducing multiscale feature fusion allows the model to capture detailed and global information.

**Expanding the dataset size:** To increase the generalization ability of the model, a larger dataset can be constructed by collecting more thyroid nodule data [59]. At the same time, increasing data diversity helps the model to better adapt to actual clinical data.

**Reduce computing complexity and memory requirements:** In resource-constrained scenarios, lightweight model structures can be developed to reduce computational complexity and memory requirements [60]. In addition, model compression and distillation techniques can be used to maintain performance while reducing resource requirements.

**Multi-modal data fusion:** As different imaging techniques can provide different information, multimodal data (e.g., ultrasound, CT, MRI, etc.) can be fused [61] further to improve the localization and classification performance of thyroid nodules.

**Broadening the scope of application:** ThyroidNet can be applied to other medical image localization and classification tasks, such as lung nodules and liver tumors, to explore the universality and adaptability of the model in different domains.

In conclusion, ThyroidNet performs well in the localization and classification of thyroid nodules, providing robust support for clinical diagnosis and treatment. By continuously optimizing the model structure, expanding the dataset size, reducing the computational complexity and memory requirements, and expanding the application domain, ThyroidNet is expected to provide constructive solutions for more medical image analysis tasks in the future.

## 6 Conclusion

This paper presents ThyroidNet, an innovative deep learning-based thyroid nodule localization and classification method. ThyroidNet unifies the localization and classification tasks by incorporating TransUnet and multitask learning, thus facilitating efficient and accurate thyroid nodule detection. The experimental section details the design, evaluation metrics, and performance analysis of ThyroidNet. The experimental findings show that ThyroidNet outperforms other techniques in terms of thyroid nodule localization and classification accuracy.

**This paper’s primary contributions are as follows:**

ThyroidNet, a groundbreaking deep learning approach, has been devised to locate and classify thyroid nodules precisely. By integrating TransUnet and multitask learning, ThyroidNet achieves significant performance improvements in thyroid nodule localization and classification tasks.

We are enhancing ThyroidNet’s performance through data augmentation strategies, network structure refinements, and loss function (DualLoss) design, resulting in a model with robust generalization capabilities suitable for handling real-world clinical data.

A comprehensive comparison of ThyroidNet with other methods, including U-Net-based, TransUnet-based, traditional image segmentation methods, FCN, Mask R-CNN, SegNet, and DeepLab, was conducted, revealing the superior performance of ThyroidNet in terms of accuracy in the localization and classification of thyroid nodules. Furthermore, examining the variation in performance across various categories of thyroid nodules offers valuable insights that can be utilized to enhance and refine the model in future iterations.

An exploration of ThyroidNet’s potential value in clinical practice, highlighting its ability to reduce the risk of misdiagnosis and missed diagnoses, increase diagnostic efficiency, and support the formulation of personalized treatment plans.

Despite the impressive results of ThyroidNet in the localization and classification of thyroid nodules, there are still limitations and challenges to be addressed. Future research may include optimizing the model structure, increasing the dataset size, reducing computational complexity and memory requirements, and exploring applications in other medical imaging domains. By continuously refining and expanding its capabilities, ThyroidNet is expected to provide valuable solutions for various medical image analysis tasks and advance the medical imaging field by continuously refining and expanding its capabilities.

## Figures and Tables

**Table 1:** The localization and classification performance comparison of different models

	Dice	Accuracy	Precision	Recall	F1
ThyroidNet	0.957	0.950	0.984	0.971	0.976
U-Net	0.918	0.911	0.918	0.916	0.917
TransUnet	0.942	0.935	0.946	0.942	0.944
Traditional	0.782	0.796	0.804	0.773	0.788
FCN	0.901	0.896	0.905	0.900	0.902
Mask R-CNN	0.936	0.930	0.942	0.940	0.939
SegNet	0.875	0.872	0.879	0.871	0.875
DeepLab	0.922	0.916	0.928	0.916	0.922

**Table 2:** The localization and classification performance comparison of different data sets

	Dice	Accuracy	Precision	Recall	F1
Private Dataset	0.957	0.950	0.984	0.971	0.976
TirAds	0.937	0.933	0.940	0.938	0.937
MASS	0.928	0.923	0.934	0.931	0.933
MIMT	0.914	0.908	0.918	0.912	0.914
CameLyon-16	0.936	0.932	0.940	0.937	0.938
MEDI	0.942	0.937	0.950	0.940	0.945
BimCv	0.894	0.887	0.901	0.893	0.898
ImageCLEFmed	0.927	0.922	0.934	0.930	0.930

**Table 3:** Performance comparison under different strategies, adjustments, and designs

Model	Dice	Accuracy	Precision	Recall	F1
ThyroidNet (No augmentation)	0.930	0.925	0.935	0.931	0.933
ThyroidNet (No MT)	0.942	0.935	0.946	0.942	0.944
ThyroidNet (Original loss)	0.948	0.940	0.958	0.950	0.954
ThyroidNet	0.957	0.950	0.984	0.971	0.976

**Table 4:** The performance difference of different types between thyroid nodules

	Dice	Accuracy	Precision	Recall	F1
Benign possible	0.932	0.960	0.970	0.958	0.961
Low suspicious	0.972	0.950	0.977	0.973	0.973
Moderately suspicious	0.966	0.900	0.984	0.982	0.982
Highly suspicious	0.948	0.930	0.965	0.980	0.969
Highly malignant	0.953	0.960	0.971	0.981	0.971

## Data Availability

The data that support the findings of this study are available on request from the corresponding author, upon reasonable request.
